# SPECT-derived myocardial perfusion and viability as predictors of response to left bundle branch pacing for cardiac resynchronization therapy

**DOI:** 10.1093/europace/euaf301

**Published:** 2025-12-02

**Authors:** Zhongwei Jiang, Zhihui Hou, Xiao Yu, Zhongqiang Zhao, Ju Bu, Chunxiang Li, Gang Yang, Cheng Wang

**Affiliations:** Department of Cardiology, The First Affiliated Hospital with Nanjing Medical University, No. 300 Guangzhou Road, Nanjing, Jiangsu 210029, China; Department of Cardiology, The First Affiliated Hospital with Nanjing Medical University, No. 300 Guangzhou Road, Nanjing, Jiangsu 210029, China; Department of Otolaryngology-Head and Neck Surgery, Shanghai Sixth People's Hospital Affiliated to Shanghai Jiao Tong University School of Medicine, Shanghai 200030, China; Department of Cardiology, The First Affiliated Hospital with Nanjing Medical University, No. 300 Guangzhou Road, Nanjing, Jiangsu 210029, China; Department of Cardiology, The First Affiliated Hospital with Nanjing Medical University, No. 300 Guangzhou Road, Nanjing, Jiangsu 210029, China; Department of Cardiology, The First Affiliated Hospital with Nanjing Medical University, No. 300 Guangzhou Road, Nanjing, Jiangsu 210029, China; Department of Cardiology, The First Affiliated Hospital with Nanjing Medical University, No. 300 Guangzhou Road, Nanjing, Jiangsu 210029, China; Department of Cardiology, Jiangsu Provincial People’s Hospital Chongqing Hospital (Qijiang District People’s Hospital), No. 54, Tuowan Branch Road, Chongqing 401450, China; Department of Cardiology, The First Affiliated Hospital with Nanjing Medical University, No. 300 Guangzhou Road, Nanjing, Jiangsu 210029, China; Department of Cardiology, Jiangsu Provincial People’s Hospital Chongqing Hospital (Qijiang District People’s Hospital), No. 54, Tuowan Branch Road, Chongqing 401450, China

**Keywords:** Left bundle branch pacing, Cardiac resynchronization therapy, Single-photon emission computed tomography

## Introduction

Left bundle branch pacing (LBBP) has emerged as a promising approach for cardiac resynchronization therapy^1–3^ (CRT), but optimal patient selection remains unclear. Pre-procedural imaging, particularly single-photon emission computed tomography (SPECT), provides valuable insights into myocardial function and perfusion.^4,5^ This study investigates the predictive and prognostic value of SPECT parameters in LBBP for heart failure (HF) patients.

## Method

We retrospectively analysed 96 HF patients with CRT indications who underwent LBBP implantation and pre-procedural gated resting SPECT-MPI.

**Figure 1:**
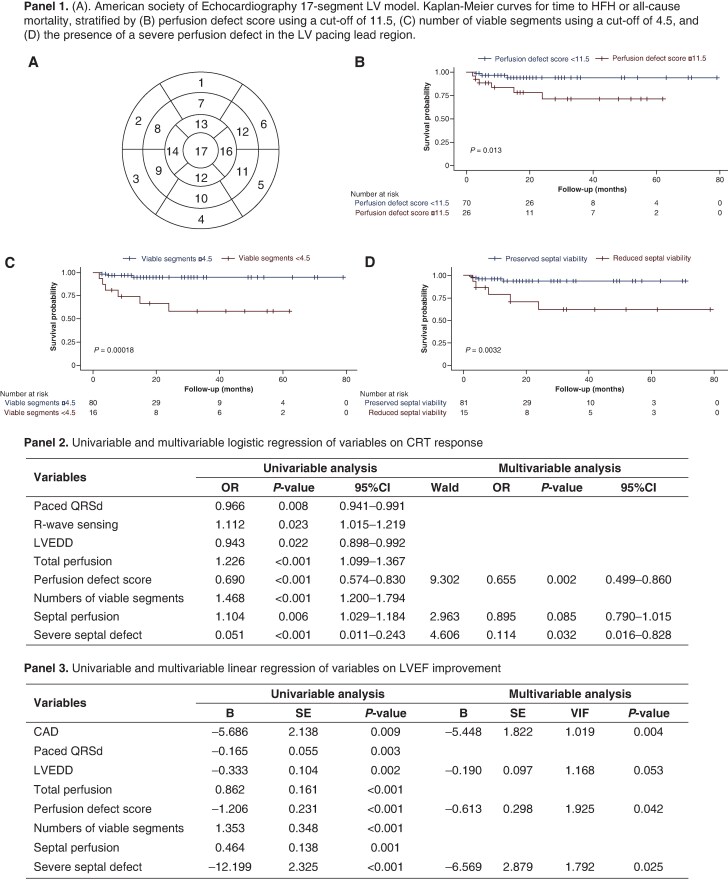
SPECT-derived myocardial perfusion and viability predict response and outcomes after LBBP-CRT. **Panel 1.** (*A*). American Society of Echocardiography 17-segment LV model. Kaplan–Meier curves for time to HFH or all-cause mortality, stratified by (*B*) perfusion defect score using a cut-off of 11.5, (*C*) number of viable segments using a cut-off of 4.5, and (*D*) the presence of a severe perfusion defect in the LV pacing lead region. **Panel 2.** Univariable and multivariable logistic regression of variables on CRT response. **Panel 3.** Univariable and multivariable linear regression of variables on LVEF improvement.

Gated resting SPECT-MPI was performed using Tc-99m sestamibi, and myocardial perfusion was automatically quantified with QPS/QGS software based on the ASE 17-segment model^6^ (*Figure 1*, panel 1A). Perfusion severity was classified as follows: Score 0 for segments with ≥75% of maximum perfusion (viable); Score 1 for 50–74% (hibernating); Score 2 for 25–49%; and Score 3 for ≤ 25% (nonviable). A total perfusion defect score was calculated as the sum of the segment scores, with severe perfusion defect defined as a tracer uptake <50% (Score 2 or 3). This criterion was specifically applied to assess whether severe defects in the LV lead implantation region (mid-septal Segments 2 and 3) correlate with the efficacy of LBBP-CRT.

LBBP was performed successfully as previously described,^7,8^ with device parameters, baseline clinical characteristics, 12-lead ECG QRS duration, and echocardiographic measurements collected.

All patients underwent routine follow-up at 6 months. LBBP response was defined as a ≥15% improvement in LVEF. Reverse remodelling was assessed by changes in LVEF, LVEDD, and LVESD. A composite outcome of HF hospitalization or all-cause mortality was also evaluated.

## Statistical analysis

Continuous and categorical variables are presented as mean ± SD or counts (percentages) and compared using t-/Mann–Whitney *U* or *χ*^2^/Fisher's exact tests, respectively. Multivariable logistic regression, linear regression, and Cox proportional hazards regression were used to assess the relationship between SPECT parameters and outcomes. The discriminative ability of SPECT parameters in predicting LBBP response was evaluated using ROC analysis, with the optimal cut-off value determined by the Youden index. Kaplan–Meier curves were used for event-free survival analysis. All analyses were performed using SPSS (version 27), with *P* < 0.05 considered statistically significant.

## Results

A total of 96 patients (mean age 65.3 years, 55.2% male) with baseline LVEF of 29.8 ± 6.7% and significant LV dilation were enrolled. SPECT revealed substantial perfusion abnormalities (defect score 10.3 ± 3.8; viable segments 7.4 ± 2.6). At 6 months, 61 patients (63.5%) responded to LBBP, with non-responders showed higher NT-proBNP, worse NYHA class, wider paced QRSd, and lower R-wave sensing. Non-responders also had higher perfusion defect scores and fewer viable segments, with severe septal perfusion defects present in 37.1 vs. 3.3% of responders (all *P* < 0.001).

Multivariable analyses identified both perfusion defect score (adjusted OR 0.655, *P* = 0.002) and severe septal defect (adjusted OR 0.114, *P* = 0.032) as independent predictors of CRT response (*Figure 1*, panel 2). These SPECT abnormalities also showed strong associations with LVEF improvement (perfusion defect score, B = −0.613, *P* = 0.042; severe septal defect, B = −6.569, *P* = 0.025, *Figure 1*, panel 3). Severe septal defects were present in 15 (15.6%) patients and were linked to markedly larger perfusion defects, fewer viable segments, and significantly poorer reverse remodelling. Patients without such defect demonstrated substantially greater LVEF improvements and larger reductions in LV dimensions at 6 months (all *P* < 0.01).

ROC analysis showed good predictive performance for perfusion defect score (AUC 0.769) and viable segments (AUC 0.744). A perfusion defect score >11.5 identified non-responders with 71.4% sensitivity and 80.3% specificity, whereas ≥5 viable segments predicted responders with 80.3% sensitivity and 68.6% specificity.

During a mean follow-up of 20.4 months, 9 patients (9.4%) were HF hospitalized. Higher perfusion defect score (HR 1.269, *P* < 0.001), severe septal defects (HR 5.249, *P* = 0.014), and fewer viable segments (HR 0.684, *P* = 0.012) were strongly associated with adverse outcomes. Kaplan–Meier curves showed significantly worse event-free survival in patients with perfusion defect score >11.5 (*Figure 1*, panel 1B), viable segments <5 (*Figure 1*, panel 1C), or severe septal defects (*Figure 1*, panel 1D).

## Discussion

This study highlights the clinical significance of SPECT-derived perfusion imaging in predicting CRT efficacy via LBBP. We found that the extent of viable myocardium and global perfusion defect burden were independently associated with reverse remodelling and long-term clinical outcomes. Notably, the location of perfusion defect, particularly severe impairment in the mid-septum, strongly diminished therapeutic efficacy.

The underlying pathophysiology may involve two distinct mechanisms. Structural defects represent irreversible scar from prior infarction, which impedes rapid Purkinje activation and renders myocardium electrically and mechanically inactive, thereby limiting resynchronization. Conversely, functional defects resulting from dyssynchrony may be ameliorated by LBBP through restoration of physiological activation. However, in the setting of advanced fibrosis or maladaptive remodelling, the potential for functional recovery remains constrained.

A key novel finding was the critical importance of septal substrate integrity. Consistent with previous CMR evidence,^9^ we demonstrated that severe mid-septal defects, the primary pacing site for LBBP, impair reliable lead capture and slow *trans*-septal conduction, leading to suboptimal electrical resynchronization and diminished haemodynamic benefit. For patients with severe septal pathology, conventional BVP may provide better resynchronization.^10^ Future studies should determine imaging-based criteria to guide the choice of optimal CRT pacing strategy for individual patients.

## Conclusion

The perfusion defect score and myocardial viability derived from SPECT were significantly associated with reverse remodelling, LBBP response, and the risk of composite cardiac events. Severe defects at mid-septum were linked to reduced CRT efficacy. Pre-procedure SPECT evaluation may provide valuable guidance in identifying patients most likely to benefit from LBBP.

## Data Availability

Data are available on demand.
